# Mentalizing and suicide reattempt: a 12-months follow-up study

**DOI:** 10.1192/j.eurpsy.2025.2346

**Published:** 2025-08-26

**Authors:** J. Andreo-Jover, B. Orgaz Alvarez, E. Suárez-Soto, P. Saiz Martinez, A. González Pinto, M. Ruiz Veguilla, A. Cebria, N. Roberto, M. Diaz Marsa, M. F. Bravo Ortiz, A. Palao-Tarrero, V. Perez-Sola

**Affiliations:** 1 Department of Psychiatry, Universidad Autónoma de Madrid (UAM); 2 Hospital La Paz Institute for Health Research (IdiPAZ),; 3 Department of Psychiatry, Clinical Psychology and Mental Health, La Paz University Hospital; 4 Departamento de Medicina Legal, Psiquiatría y Patología, Universidad Complutense de Madrid, Madrid; 5 Department of Psychiatry, Universidad de Oviedo, Oviedo; 6 Hospital Santiago de Araba, Alava; 7 Hospital Virgen del Rocio, Sevilla; 8 Hospital Parc Tauli; 9 Bipolar Disorders Unit, Hospital Clinic, Institute of Neurosciences, University of Barcelona, IDIBAPS, Barcelona; 10 Hospital Clinico San Carlos; 11 Department of Psychiatry, Clinical Psychology and Mental Health, La Paz University Hospital, Madrid; 12 Institut de Neuropsiquiatria i Addiccions (INAD), Parc de Salut Mar, Barcelona, Spain

## Abstract

**Introduction:**

Hypomentalizing may contribute to heightened social withdrawal and it has been related with an increased risk of and suicide attempt (SA). Although certain studies have identified a relationship between hypomentalizing and suicidal behavior, research on follow-up remains limited.

**Objectives:**

This study aims to examine the relationship between suicide reattempt and the progression of mentalizing within 12-months.

**Methods:**

Our study included a cohort of 1,374 patients who committed a SA. We conducted assessments at the baseline and at a 12-months follow-up. We measured mentalizing using the RFQ-8, and evaluations of suicidal ideation and behavior employing the CSRSS. Demographics, clinical characteristics, and mentalizing were subjected to comparative analysis using the T-student and Chi-square tests.

**Results:**

A total of 310 participants committed a suicide reattempt in the follow-up period. Our results showed that reattempt group were significantly younger, more presence of female gender, suicidal ideation and planning, more previous SA, and higher hypomentalizing means.

**Image 1:**

**Figure:**
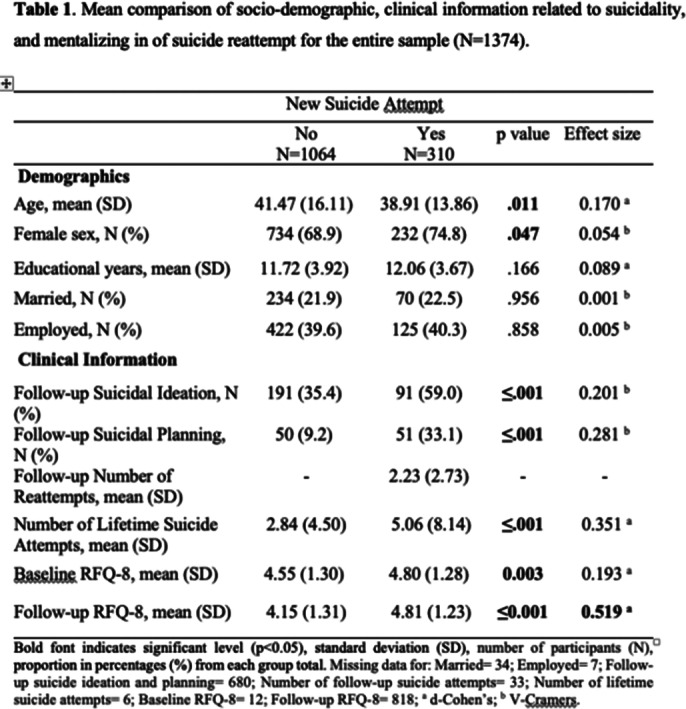

**Conclusions:**

Social cognition may play a crucial role in the suicide reattempt risk. Future research on the association between social cognition and suicidal behavior could help elucidate the associated factors and identify potential therapeutic actions.

**Disclosure of Interest:**

None Declared

